# Eight weeks of dry dynamic breath-hold training results in larger spleen volume but does not increase haemoglobin concentration

**DOI:** 10.3389/fphys.2022.925539

**Published:** 2022-10-07

**Authors:** Kun Yang, Wen-Bin Wang, Ze-Hua Yu, Xiao-Lan Cui, Zhang-Biao Yu, Yi Jiang, Jin-Fei Gou, Meng-Meng Du

**Affiliations:** ^1^ School of Physical Education, Guizhou University, Guiyang, Guizhou, China; ^2^ Graduate School, Guangzhou Sport University, Guangzhou, Guangdong, China

**Keywords:** apnoea, breath-holding, spleen size, haemoglobin, red blood cells, immunity

## Abstract

**Purpose:** It has previously been reported that repeated exposure to hypoxia increases spleen size and haemoglobin (HGB) level and recent reports on the effect apnoea has on spleen size and haematological parameters are contradictory. Therefore, this study aims to evaluate the effect apnoea training has on spleen size and haematological parameters.

**Methods:** The breath-holding (BH) group was comprised of 12 local student-athletes with no BH exercise experience who performed BH jogging and BH jumping rope dynamic apnoea protocols, five times weekly for 8 weeks. The BH event duration was progressively increased as the apnoea tolerance of the athletes improved (20 to 35 s). The same training task was performed by the control group (*n* = 10) without BH. Spleen sizes were measured with an ultrasound system and a complete blood cell analysis was performed on the median cubital venous blood.

**Results:** Spleen volume in the BH group increased from 109 ± 13 ml to 136 ± 13 ml (*p* < 0.001), and bulky platelets decreased from 70.50 ± 5.83 to 65.17 ± 5.87 (*p* = 0.034), but no changes were recorded for erythrocytes (*p* = 0.914), HGB (*p* = 0.637), PLTs (*p* = 0.346) and WBC (*p* = 0.532). No changes were recorded for the control group regarding spleen size or haematological parameters.

**Conclusion:** Eight weeks of dry dynamic apnoea training increased spleen size and decreased the number of circulating bulky platelets in the athletes who were assessed in this study. However, the baseline RBC counts and HGB levels of the athletes were not altered by the training programme.

## 1 Introduction

Mammalian oxygen conservation mechanisms are activated rapidly during apnoea or diving (Kooyman et al., 1981; Schagatay et al., 2007), inducing a cardiovascular diving reflex that is characterised by heart rate deceleration, vasoconstriction and blood redistribution (Elsner and Gooden. 1983; Qvist et al., 1986), effectively slowing oxygen consumption and extending apnoea duration (Gooden 1994; Schagatay et al., 2001).

Another effective mechanism through which mammals respond to hypoxia is splenic contraction (Hurford et al., 1996; Cabanac et al., 1997). The spleen is a major blood reservoir in mammals (Anderson and Rogers., 1957; Guntheroth and Mullins., 1963; Espersen et al., 2002) and during strenuous exercise or apnoea, splenic contraction pumps stored concentrated red blood cells (RBCs) into circulation (Koga, 1979; Laub et al., 1993; Stewart and McKenzie. 2002; Bakovic et al., 2005; Richardson et al., 2009; Bakovic et al., 2013). This increases blood oxygen storage and oxygen-uptake kinetics (Thomas and Fregin, 1981; Longhurst et al., 1986; Wagneft et al., 1995; Schagatay et al., 2001; Brijs et al., 2020; Holmström et al., 2021). For example, hooded seals release oxygenated haemoglobin (HGB) from the spleen into circulation, which can increase dive duration by approximately 105 s, and harp seals can increase drive duration by 80 s (Cabanac et al., 1997). Splenectomy results in the loss of this ability (Persson et al., 1973; Thomas and Fregin, 1981; Baković et al., 2005; Bakovic et al., 2013; Brijs et al., 2020; Joyce and Axelsson. 2021).

Researchers have discovered that repeated low-oxygen exposure and long-term hypoxia cause splenic expansion (Bouten et al., 2019; Holmström et al., 2019; Lodin-Sundström et al., 2021). Under hypoxia, hypoxia-inducible factor (HIF-2) activates the erythropoietin (EPO) transcription gene while promoting renal cortex EPO-expressing cells as a means of producing EPO, thereby stimulating the production of erythrocytes and HGB (Tsuchiya et al., 1997; Warnecke et al., 2004; Jelkmann 2011).

Extensive studies of splenic and haematological reactions after apnoea have already been performed in the literature (Shephard et al., 2016; Elia et al., 2021; Pernett et al., 2021; Nordine et al., 2022). It has been proven that long-term static apnoea training increases both spleen volume and HGB (Bouten et al., 2019). As far as we are aware, no previous studies have reported the longitudinal effects of dry dynamic apnoea training on splenic size and haematological parameters. Dry dynamic apnoea has physiological demands that are significantly different to static apnoea (Bergman et al., 1972; Elia et al., 2021c; Nordine et al., 2022). Under dry dynamic apnoea conditions, myocardial and skeletal muscle oxygen consumption increases, heart rate becomes faster and acute conflict occurs between the oxygen conservation mechanism of the body and skeletal muscle oxygen demand (Elia et al., 2019; Elia et al., 2021c; Nordine et al., 2022). It can be reasonably assumed that dry dynamic apnoea training protocols provide greater hypoxic stimulation than static apnoea (Elia et al., 2019; Elia et al., 2021c; Nordine et al., 2022), but further research is required to confirm this.

Therefore, the aim of this study is to investigate the effect 8 weeks of dry dynamic breath-holding (BH) training has on spleen size and haematological parameters among student-athletes. As the spleen is a significant reservoir for immune cells, relevant parameters are included as indicators for assessing whether BH training induces immune function changes that have not been previously performed. It is hypothesised that 8 weeks of dry dynamic BH training increases spleen volume, RBC counts and HGB levels, but has no impact on immune function.

## 2 Materials and methods

### 2.1 Ethical aspect

This study is in full compliance with the World Medical Association Declaration of Helsinki and received ethical approval from the Guizhou University subcommittee of Human Medicine Experimental Ethics (No: HMEE-GZU-2022-T002). All volunteers were fully informed of any potential risks presented by this study and all provided written informed consent before their participation. The parents or guardians of subjects under 18 years of age signed informed consent forms on the behalf of their children.

### 2.2 Study design

**FIGURE 1:**
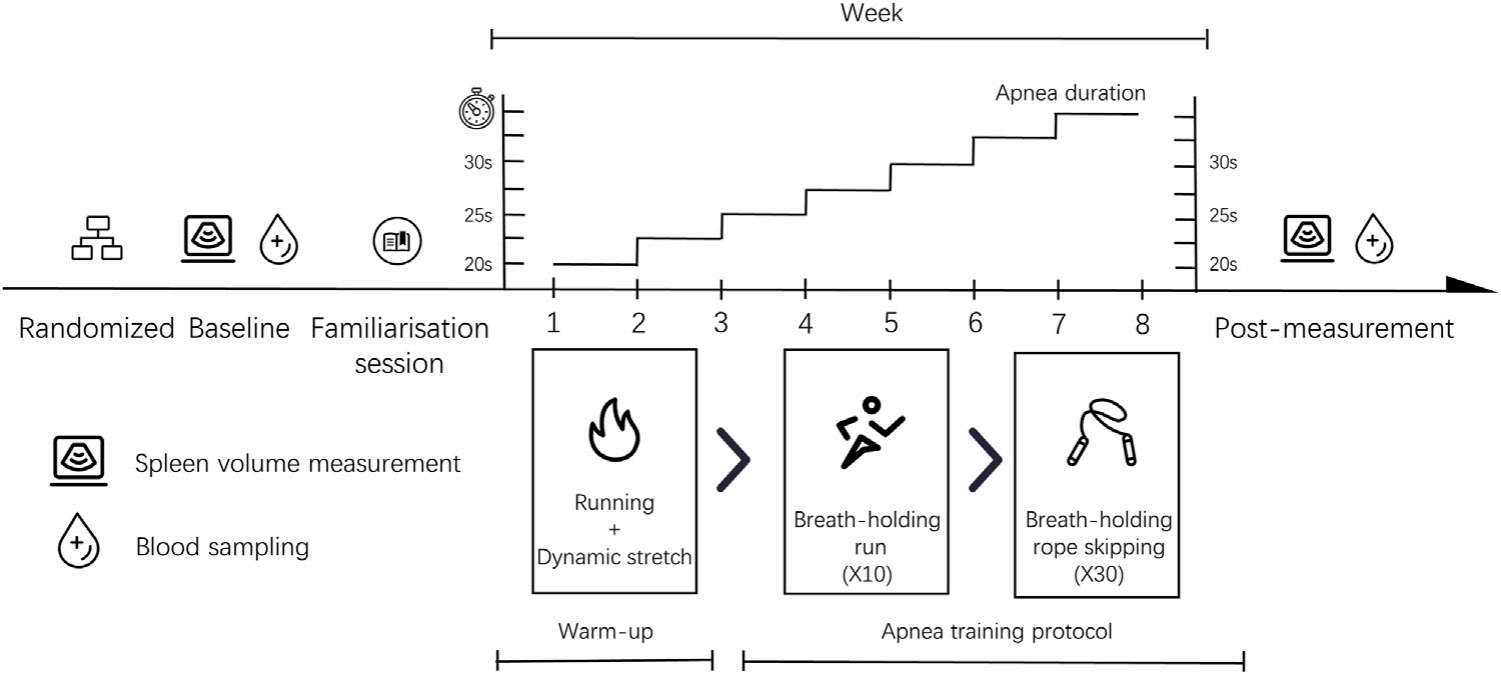
Study design.

The study was performed using a 2-arm randomised controlled experimental design for 8 weeks and involved five consecutive training sessions each week. The training consisted of two phases and lasted a total time of approximately 60 min. The first phase involved a 15-min warm-up, including a 10-min run and 5 minutes of stretching exercises. The second phase was dynamic apnoea training that lasted for approximately 45 min. Participants were subjected to baseline spleen volume measurement and venous blood sampling prior to the study. This was followed by a familiarisation session. The spleen size and venous blood samples of all volunteers were collected within 48 h of completion of the training. The same researchers were responsible for the management of the entire study. The experimental procedure can be seen in Figure 1.

### 2.3 Participants

Twenty six local student-athletes from Guizhou University were recruited to participate in this study. The volunteers were stratified according to gender prior to commencement of the study and then completely randomised into groups within each stratum using a computer-generated random number sequence. They were assigned to either the BH group or the control (Con) group.

Four volunteers (BH group, two men; Con group, one man and one woman) were excluded from the study as a result of withdrawal or low attendance. Other volunteers fully completed the training plans.

Inclusion criteria included members of the local population, aged 17–24 years, physically and mentally healthy and a willingness to attend long-term training sessions. Exclusion criteria included suffering from recent sports injuries, regular drug-taking, genetic disease or family disease history, having donated blood or smoked in the last 3 months, participation in BH-related sports (including free diving or synchronised swimming) and suffering from obstructive sleep apnoea syndrome. All volunteers were asked to maintain the same training level as before the study and not to donate blood, travel at high altitude or participate in any other studies. They were also advised to strictly adhere to the prescribed routine and informed that they could withdraw from the study at any time.

## 3 Experimental procedures

### 3.1 Preliminary measures

Anthropometrics, spleen quantification and blood sampling were conducted at Guiyang Huaxi District People’s Hospital. Weight and height measurements were taken using the X-Scan Plus II (JAWON, South Korea). Blood pressure and heart rate were measured using an electronic blood pressure monitor (YE-680, Yuwell, Suzhou, China). It was requested that all subjects avoid any vigorous activity and refrain from the consumption of caffeinated and alcoholic beverages 24 h prior to the test. In addition, they were required to abstain from food and water for 8 h before the measurement.

### 3.2 Spleen imaging

Tests were conducted in the morning. All volunteers were asked to rest on their backs for 20 min upon arrival at the imaging centre (∼25°C) and this was followed by a spleen scan. Subjects were positioned in the right lateral recumbent position, exposing the left lumbar and abdomen. At this time point, they were able to breathe freely. Maximum spleen length (L), maximum width (W) and maximum thickness (T) were all measured by an experienced physician (Wang*) using a colour ultrasound diagnostic system (Philips Affiniti 70W, Amsterdam Dutch). The scan lasted for 8–10 min and the results were documented and evaluated by another physician.

**FIGURE 2:**
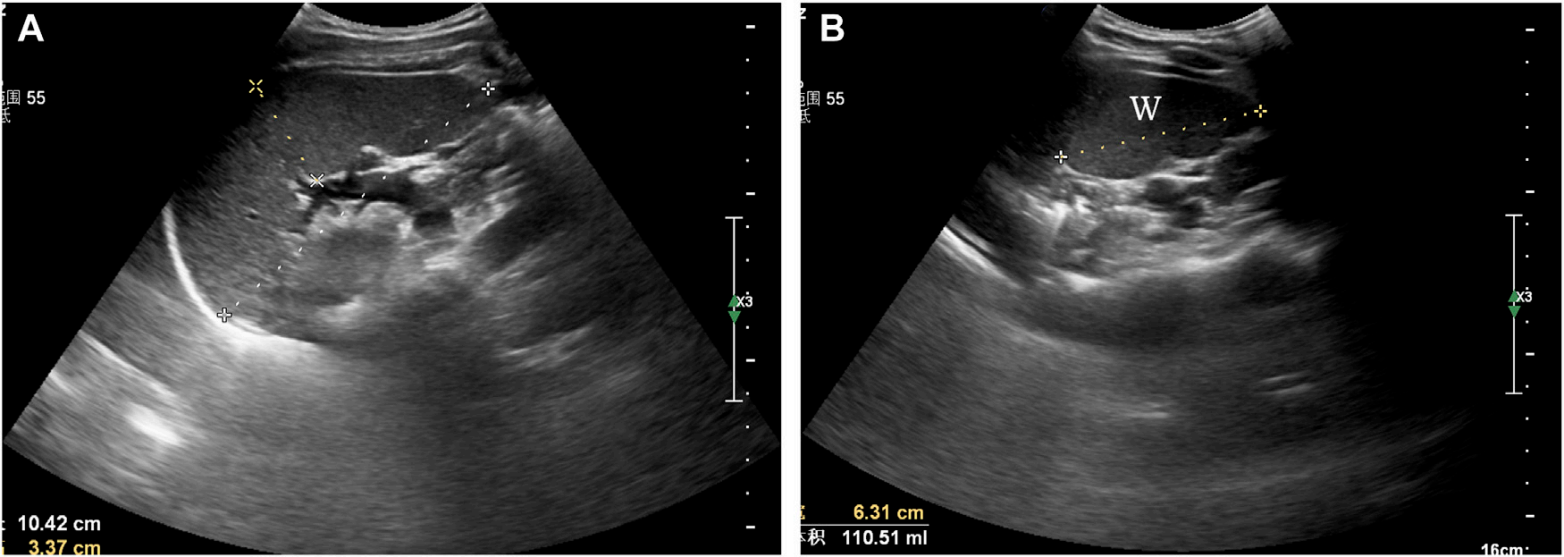
Ultrasonographic assessment of spleen volume. **(A)** maximum spleen length (L) and thickness (T), **(B)** maximum spleen width (W).

Figure 2A shows the maximum spleen length and thickness. Length was measured as the greatest overall dimension and thickness was measured as the shortest distance between the hilum and the outer convex surface of the spleen. Measurements were taken as perpendicular to each other as was possible. Figure 2B shows the maximum spleen width. Maximum width was measured as the greatest overall dimension. Spleen volume was calculated using the long ellipsoid formula: Spleen volume (ml) = 0.523 × (L × T × W).

### 3.3 Blood sample and analysis

A 2–3 ml blood sample was collected from the median cubital vein in an ethylenediaminetetraacetic acid (EDTA) vacuum blood collection tube. It was stored at 2–8°C, protected from shock and examined within 2 h of collection. The collected blood samples were analysed using the Mindray BC-6800 Plus, BC-5180 CRP (Shenzhen, China) fully automated haematology analyser.

### 3.4 Familiarisation session

The researchers conducted a familiarisation session within 48 h of completion of the baseline measurements. All volunteers were provided with a detailed guide to the precautions during BH. They were informed to avoid hyperventilation and excessive air inhalation before holding their breath, concentrate on the BH task and not to swallow or exhale any gas during the BH. They were also guided to familiarise themselves with the entire experiment procedure and practice it until they were able to complete all training tasks correctly and successfully.

### 3.5 Apnoea training protocol

The training is conducted on a plastic track with cushioning technology, which can effectively reduce the risk of injury caused by falls. The dynamic apnoea training protocol involved 10 BH sessions of jogging and 30 repeats with a jump rope. A fixed BH duration and an escalating load model were used in the study. Volunteers were asked to achieve the 20-s BH goal during the first week. The average increase was 2.5 s per week and this peaked at 35 s in week 8. Exercise intensity was 60%–70% of the maximum heart rate load. Volunteers inhaled approximately 70%–85% of their maximum lung capacity before holding their breath. During BH sessions, the volunteers wear nose clips. In addition, finger clip pulse oximetry (Wellday MD300C23; Jiang Xi, China) was used for monitoring the heart rate and blood oxygen of participants during the training period. The device dynamically generates real-time SPO2 values within 8 s, which are read and recorded by the researcher.

#### 3.5.1 Warm-up

In order to minimise the impact warm-up would have on the results, all volunteers performed the same warm-up routine in this study, involving 10 min of jogging and 5 min of dynamic stretching exercises. Exercise intensity was approximately 65%–75% of maximum heart rate. The dynamic stretching protocol was specifically designed to enable volunteers to adequately activate critical regions, including shoulders, elbows and knees. Injury potential was minimised as much as possible during the training.

#### 3.5.2 Apnoea protocol

##### 3.5.2.1 Breath-holding run

A researcher drove a small electric vehicle with a fixed speed function and asked participants to run anticlockwise around a track at a constant speed of ∼2.35 m/s. When all volunteers were ready and wore nose clips, the researchers signalled them to hold their breath using a verbal command and timed them using a stopwatch. During the final 10 s of apnoea duration, the researchers gave an oral countdown and offered verbal encouragement. Once the apnoea was completed, the researchers told the volunteers to stop holding their breath and to continue their run. At this point, volunteers were allowed to remove the nose clip breathe freely and continue to the next training after 30 s. This process was repeated a total of 10 times. The Con group performed the same training task without BH and the researchers monitored them during the training period. After completing the training, volunteers were allowed a relaxation break of 2 min.

##### 3.5.2.2 Breath-holding jump rope

When all volunteers were ready and wore nose clips, the researchers signalled them to hold their breath using a verbal command and timed them using a stopwatch. During the final 10 s of apnoea duration, the researchers gave an oral countdown and offered verbal encouragement. At the end of the apnoea duration, the volunteers were told to stop holding their breath and were allowed to breathe freely and relax. After 30 s of rest, the next session was performed immediately. This process was repeated a total of 30 times. The control volunteers performed the same training task without BH and researchers monitored them during the training period.

#### 3.5.3 Termination of experimental criteria

Training was terminated temporarily if any volunteer experienced dizziness, blurred vision, tinnitus, or nausea or had a pulse oximetry (SpO2) level of <60. In addition, an emergency response team of two trained researchers was in place to address any accidents that may occur during the training. The training site was near a hospital, meaning that volunteers could receive timely medical assistance in case of accident.

### 3.6 Statistical analysis

All data analysis and graph construction were performed using IBM SPSS Statistics 26 (Armonk, NY: IBM Corp, United States) and Origin9.1 (OriginLab, Northampton MA, United States) software. Shapiro-Wilk test was applied for assessing the normal data distribution. Levene’s test was employed to test for homogeneity of variances. Repeated two-way ANOVA tests were applied as a means of assessing the differences between the resting baseline measurements and other data collection time points. Bonferroni was employed to perform multiple comparisons. Pearson’s correlation coefficients were applied for determining inter-variable relationships. Mann-Whitney *U*-test and Wilcoxon signed-rank test were used as non-parametric tests in cases where the data variance was not homogeneous or non-normally distributed. *p* < 0.05 was considered as an indicator of statistical significance. All data is presented as the mean ± standard error (SE) or 95% confidence interval (CI).

## 4 Results

### 4.1 Subjects

Table 1 shows there to be no significant differences between the groups of volunteers regarding age, height, weight, heart rate or blood pressure level. No blurred vision, tinnitus or nausea were detected during training and relatively few volunteers exhibited signs of mild dizziness at the start of the training period.

**TABLE 1:** Anthropometric characteristics of participants.

	BH group (*n* = 12, *M* = 6)	Control group (*n* = 10, *M* = 5)	*p*
	Mean ± SE	Mean ± SE	
Age (years)	21 ± 0	20 ± 0	0.327
Height (cm)	170 ± 3	174 ± 3	0.389
Body mass (kg)	59 ± 3	59 ± 3	0.999
BMI	20.3 ± 0.5	19.5 ± 0.5	0.254
BPM	63 ± 1	65 ± 1	0.215
sBP (mmHg)	109 ± 2	105 ± 3	0.418
dBP (mmHg)	66 ± 1	67 ± 2	0.582

Note: *denotes significance (*p* < 0.05).

### 4.2 Spleen volume

**FIGURE 3:**
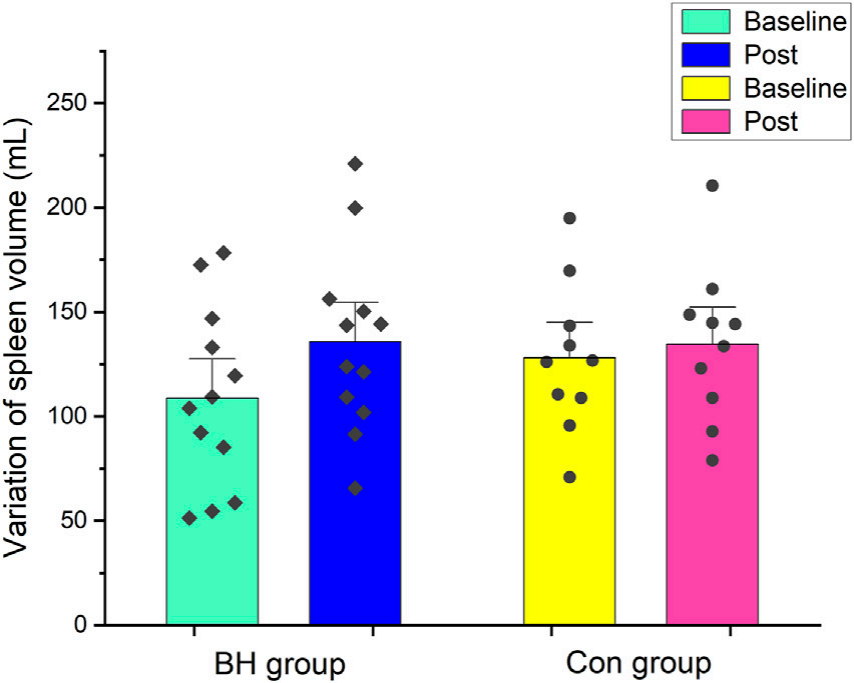
Mean (±SE) spleen volume (ml) variation from baseline to after 8 weeks in BH group and Con group.

Figure 3 shows spleen size changes in both groups after training. After 8 weeks interventional training, spleen size increased from 109 ± 13 ml (95% CI: 81–136 ml) to 136 ± 13 ml (95% CI: 108–164 ml) in the BH group (+24.72%, *p* < 0.001). No difference was observed before (128 ± 11 ml, 95% CI: 102–154 ml) or after training (135 ± 12 ml, 95% CI: 108–161 ml) in the Con group (*p* = 0.375).

**FIGURE 4:**
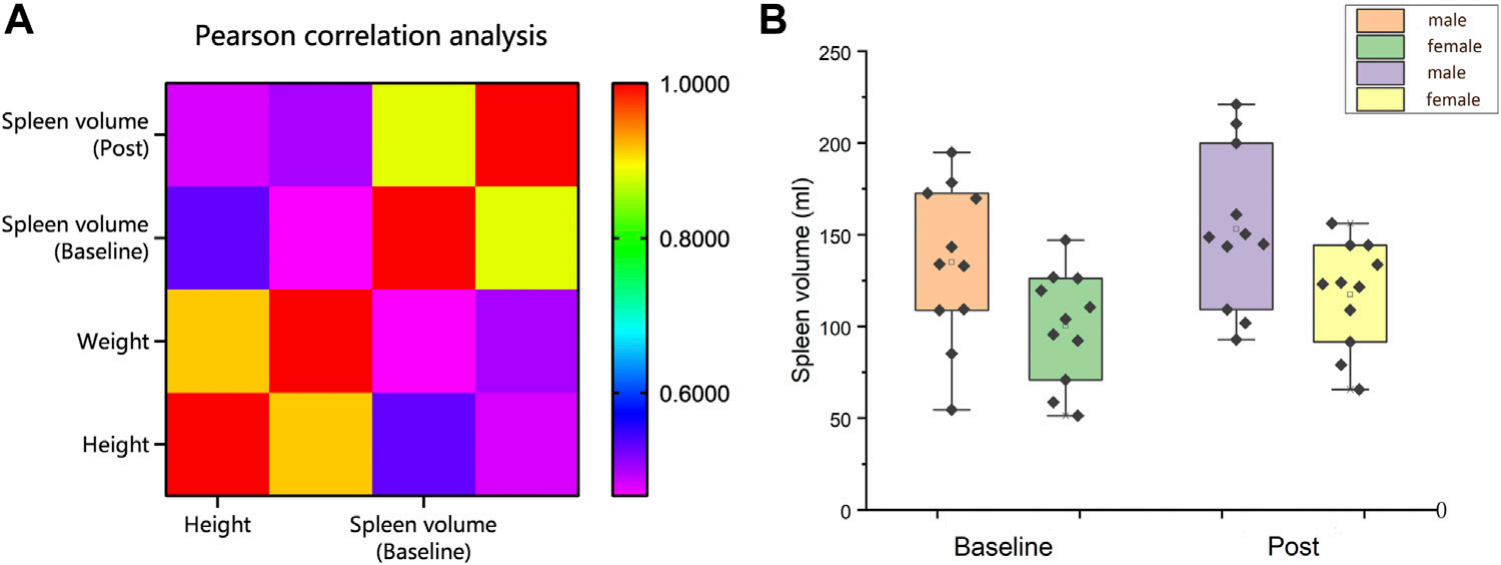
**(A)** Correlation analysis of height and weight with spleen size; **(B)** Spleen volume (ml) comparison between male and female before and after training (Mean ± SE).

Pearson’s correlational analysis found there to be a moderate correlation between spleen size and height (Baseline: *r* = 0.530, *p* = 0.011; Post *r* = 0.483; *p* = 0.023) and weight (Baseline: *r* = 0.467, *p* = 0.029; Post: *r* = 0.502; *p* = 0.017) among 22 volunteers (Figure 4A). In this study, female spleen sizes were found to be significantly smaller than male spleen sizes (Baseline: 100 ± 9 ml vs. 135 ± 13 ml, *p* = 0.041; Post: 111 ± 9 ml vs. 15 ± 13 ml, *p* = 0.033; Interaction effect: gender * time, *p* = 0.762) (Figure 4B).

### 4.3 Erythrocytes and haemoglobin

Table 2 shows RBCs, HGB level and related derived index changes following training. No changes in RBCs (*p* = 0.914), HGB (*p* = 0.637), HCT (*p* = 0.511), MCH (*p* = 0.501) or RDW-CV (*p* = 0.595) were recorded in the BH group following training. MCV decreased from 91.93 ± 1.27 to 91.27 ± 1.48 (−0.72%, *p* = 0.048) and mean cell haemoglobin concentrations (MCHC) increased from 329.92 ± 2.05 to 334.42 ± 2.76 (+1.36%, *p* = 0.026) in the BH group following training. No significant changes were observed in the Con group.

**TABLE 2:** Variations in red blood cells, haemoglobin and related derivatives.

Item (unit)	BH group		*p*	Con group		*p*
	Baseline	8 weeks post		Baseline	8 weeks post	
RBC	4.88 ± 0.14	4.87 ± 0.12	0.914	5.07 ± 0.12	5.06 ± 0.11	0.813
HGB	147.7 ± 4.3	148.4 ± 4.9	0.637	149.5 ± 5.3	149.7 ± 6.2	0.908
HCT	45 ± 1	44 ± 1	0.511	45 ± 1	45 ± 2	0.830
MCV	92 ± 1	91 ± 1*	0.048	89 ± 2	89 ± 2	0.629
MCH	30 ± 1	30 ± 1	0.501	30 ± 1	30 ± 1	0.832
MCHC	330 ± 2	333 ± 3*	0.026	330 ± 3	331 ± 3	0.392
RDW-CV	12.8 ± 0.2#	12.9 ± 0.2	0.595	13.8 ± 0.4#	13.4 ± 0.2	0.064

Notes: RBC denotes red blood cell count (10^12^/L); HGB denotes haemoglobin (g/L); HCT denotes haematocrit (%); MCV denotes mean corpuscular volume (fL); MCH denotes mean corpuscular haemoglobin (pg); MCHC denotes mean corpuscular haemoglobin concentration (g/L); RDW-CV denotes coefficient variation of red blood cell volume distribution width (%). *A significant difference in baseline values (*p* < 0.05); ^#^a significant difference between groups (*p* < 0.05).

### 4.4 Blood PLTs

Table 3 shows the changes in PLT-related indicators following training. No changes in PLTs (*p* = 0.346), mean PLT volume (MPV) (*p* = 0.052), platelet distribution width (PDW) (*p* = 0.511) or plateletcrit (PCT) (*p* = 0.100) were recorded in the BH group following training. The number of bulky platelets (P-LCC) decreased from 70.50 ± 5.83 to 65.17 ± 5.87 (−7.57%, *p* = 0.034) in the BH group following training, while no significant changes were observed in the Con group.

**TABLE 3:** Variation of platelet-related indicators.

Item (unit)	BH group		*p*	Con group		*p*
	Baseline	8 weeks post		Baseline	8 weeks post	
PLT	252.83 ± 12.39	246.67 ± 12.18	0.346	245.40 ± 11.27	244.90 ± 15.36	0.944
MPV	10.4 ± 0.4	10.1 ± 0.4	0.052	9.9 ± 0.3	9.9 ± 0.2	0.441
PDW	16.3 ± 0.1	16.3 ± 0.1	0.999	16.1 ± 0.1	16.2 ± 0.1	0.310
PCT	0.26 ± 0.01	0.25 ± 0.01	0.100	0.24 ± 0.01	0.24 ± 0.01	0.999
P-LCC	70.50 ± 5.83	65.17 ± 5.87*	0.034	62.00 ± 4.70	62.50 ± 4.75	0.847

Notes: PLT denotes platelet count (10^9^/L); MPV denotes mean platelet volume (fL); PDW denotes platelet distribution width (%); PCT denotes plateletcrit (%); P-LCC denotes platelet large cell counts (bulky platelets) (10^9^/L). *A significant difference in baseline values (*p* < 0.05); ^#^A significant difference between groups (*p* < 0.05).

Pearson’s correlation analysis found the decrease in PLTs (6.17) and P-LCC (6.60) to have a significant correlation in the BH group following training (*r* = 0.721, *p* = 0.008).

### 4.5 Immune cells

Table 4 presents the changes in the immune-cell parameters after training. No changes in WBC (*p* = 0.532), neutrophil counts (NEU) (*p* = 0.933), monocyte counts (MON) (*p* = 0.097), eosinophil counts (EOS) (*p* = 0.250), and basophile counts (BAS) (*p* > 0.999) were recorded after training in the BH group. Lymphocyte counts (LYM) increased from 1.89 ± 0.11 to 2.20 ± 0.13 (+16.40%, *p* = 0.014) in the BH group after training. No significant changes were noted in the Con group.

**TABLE 4:** Variations of immune cell parameter.

Item (unit)	BH group		*p*	Con group		*p*
	Baseline	8 weeks post		Baseline	8 weeks post	
WBC	5.55 ± 0.42	5.96 ± 0.37	0.532	6.09 ± 0.59	6.42 ± 0.77	0.649
NEU	3.21 ± 0.40	3.27 ± 0.34	0.933	3.69 ± 0.58	3.99 ± 0.90	0.708
LYM	1.89 ± 0.11	2.20 ± 0.13*	0.014	1.91 ± 0.67	1.95 ± 0.18	0.761
MON	0.34 ± 0.04	0.39 ± 0.02	0.097	0.33 ± 0.02	0.35 ± 0.02	0.627
EOS	0.09 ± 0.01	0.07 ± 0.01	0.250	0.14 ± 0.03	0.11 ± 0.03	0.294
BAS	0.02 ± 0.00	0.02 ± 0.00	0.999	0.03 ± 0.00	0.03 ± 0.00	0.999

Notes: WBC denotes white blood cell count (10^9^/L); NEU denotes neutrophil counts (10^9^/L); LYM denotes lymphocyte counts (10^9^/L); MON denotes monocyte counts (10^9^/L); EOS denotes eosinophil counts (10^9^/L); BAS denotes basophile counts (10^9^/L). *A significant difference with baseline values (*p* < 0.05); ^#^A significant difference between groups (*p* < 0.05).

## 5 Discussion

This study evaluated the effects dry dynamic apnoea training has on spleen size and haematological parameters among athletes. The primary findings showed that 8 weeks of dynamic apnoea training increased spleen size, reduced the number of bulky PLTs in circulation and showed no significant changes in HGB, RBC, PLT or WBC.

The spleen volumes of volunteers were evaluated using sonography. The techniques and standards that are used for the determination of spleen volume by ultrasonography have been presented in previous studies (Koga and Morikawa. 1975; Rezai et al., 2011). Sonography is a quick, easy, inexpensive and relatively accurate examination method and it presents no risk of radiation exposure (Yetter et al., 2003; Natsume et al., 2011). The efficacy and reliability of sonography have been determined by many studies that have compared measurements with CT scan results or autopsy results (Koga and Morikawa, 1975; Schlesinger et al., 1993; Loftuset al. 1999; Lamb et al., 2002; Yetter et al., 2003). The standard clinical ellipsoid equation (L × T × W × 0.523) was used in this study for evaluating spleen volume as it has good efficacy and is regularly used for evaluating the volume of irregularly-shaped organs including the spleen and uterus (Sauerbrei et al., 1986; Sonmez et al., 2007).

The results of this study are in accordance with those from the report by Bouten on increased spleen size in volunteers following apnoea training (Bouten et al., 2019), which reports that repeated exposure to hypoxia affects spleen size. However, no spleen volume increase was observed by Elia or Engan in their respective studies (Engan et al., 2013; Elia et al., 2021c). In comparison to the two-week training programme of Engan, the training periods of both Bouten and this study were significantly longer. Rodriguez et al. also reported a spleen volume increase of 40% after 6 weeks of travelling at high altitude (Rodríguez-Zamora et al., 2015). Therefore, it was speculated that some time-dose response may have potentially contributed to the result. More extended intervention periods and more potent stimuli are required for increasing spleen size. In a six-week study by Elia, no spleen volume increase was observed. A possible reason is that although the study extended the training periods, the training sessions (24 sessions) were fewer than those in the study by Bouten et al. (2019). Furthermore, the longer interval between weeks (3 days) may also have attenuated the training effect. Similarly, Elia et al. (2021c) noted that splenic expansion may require more prolonged periods in their study. In addition, four volunteers (BH group, two men; Con group, one man and one woman) were excluded from this study due to withdrawal or low attendance. Therefore, the BH group had a slightly smaller baseline spleen volume than the Con group. Before this, the male spleen volume is generally larger than the female spleen volume has been confirmed (Spielmann et al., 2005; Chow et al., 2016). We corrected the baseline spleen volume differences by employing baseline spleen volume as a covariate and found a significant difference in the intervention effect between the two groups (*p* = 0.023).

The mechanism for determining spleen size still remains unknown (Elia et al., 2021b). Several studies have reported spleen size to be affected by age, sex, height, weight, and ethnicity (Kaneko et al., 2002; de Bruijn et al., 2008; Hiraiwa et al., 2022). This study determined a correlation between spleen size and height (*r* = 0.530; *p* = 0.011) and weight (*r* = 0.467; *p* = 0.029). In contrast, Elia et al. reported there to be no direct correlation between spleen size and height (*r* = −0.0001, *p* = 0.995) or weight (*r* = −0.040, *p* = 0.863) in their study on a group of elite BH divers and non-divers (Elia et al., 2019). Similar results were reported in a study by Schagatay et al. (2012). This difference in results can potentially be explained by spleen size being significantly different between individuals (Elia et al., 2021b). In addition, spleen size may be affected by acquired factors including hypoxia or training (Rodríguez-Zamora et al., 2015; Bouten et al., 2019; Kaiser et al., 2022). Therefore, the occupations of subjects and the activities they perform must be adequately considered.

Ilardo et al. (2018) conducted a comparative genetic study on the outstanding Bajau divers in Southeast Asia. They found spleen size to be genetically determined without any phenotypic plasticity (Ilardo et al., 2018). However, some recent studies have suggested that spleen size may be influenced by complex interaction between genetic susceptibility and environmental exposure (Schagatay et al., 2020; Holmström et al., 2020; Bouten et al., 2019; Rodríguez-Zamora et al., 2015; Schagatay et al., 2015). For example, Sherpas who live at high altitude tend to have larger spleen volumes than those who live at lower altitude and the average spleen volume of Sherpas is larger than that of Nepalese people who live at lower altitudes (Holmström et al., 2020). In addition, it has been confirmed that the spleen has regenerative abilities (Pabst et al., 1984; Ando et al., 2004; Ibrahim et al., 2005; Petrovai et al., 2013). Researchers found that among 207 athletes in the Lanzhou region of China who engaged in different sports, 71.2% of males and 66.7% of females had enlarged spleens, while 56% of males and 34% of females had enlarged livers in comparison to those of the local population. The authors of the paper suggested that blood flow, oxygen consumption and metabolism due to high-intensity exercise can result in the spleen making changes as a means of adapting to the demands of the body. However, the study made no further distinctions based on the sports that were undertaken by the athletes (MingLi et al., 1992).

In this study, no significant changes were detected following training in both RBC and HGB, which is in accordance with the study results of Engan et al. (2013) and Elia et al. (2021c). EPO plays a crucial role in the regulation of RBC and the production of HGB (Adamson 1996; Jelkmann 2003). EPO production increases in the context of systemic hypoxia or hypoxemia. In 2008, de Bruijn et al. (2008) reported that circulating EPO concentrations increased by 16% over 3 h following a series of repeated BH events, which indicates that the erythropoietic process is enhanced (Banfi 2008). Similar observations were reported by Kjeld et al. (2015) and Elia et al. (2019). However, some researchers have conservative opinions regarding whether the increase in EPO concentration induced by BH is sufficient for stimulating an increase in erythrocyte and HGB volume (Elia et al., 2021b). For example, Engan et al. (2013) reported reticulocyte counts to increase by 15% (*p* < 0.05) following BH training, while no significant change was detected in the baseline HGB. Similar results were also reported by Elia et al. (2021c). Conversely, Bouten et al. (2020) reported a 3.3% increase in baseline HB following 8 weeks of BH training. However, it was also noted that the effect of plasma and blood volume changes in HB could not be ruled out completely. In this study, no HGB changes could be attributed for the following reasons: 1) volunteers lived at medium or high altitude for a long time (1,130 m above sea level), making them resistant to low-oxygen level; 2) baseline erythrocyte (male: 5.18, female: 4.58) and HGB levels (male: 159.33, female: 136.00) of the volunteers were already incredibly close to the upper limit of the range of normal values prior to the intervention. Therefore, the training may have been insufficient for the generation of more powerful stimuli for erythrocytes and HGB production in organisms.

It is believed that this is the first study that has noted a decrease in baseline P-LCC following apnoea training. PLT levels were not altered and the spleen is a dynamic reservoir of bulky PLTs (Bakovic et al., 2013). Exchangeable splenic PLT pool size increases as spleen size increases (*r* = 0.76, *p* < 0.001) (Wadenvik et al., 1987). When the spleen becomes enlarged, splenic blood PLT density is higher than circulating PLT density (Aster 1966). In addition, Ando et al. (2004) found there to be a distinctly negative correlation between an increase in spleen volume and circulating PLT count (*r* = −0.411, *p* = 0.045). Therefore, a potential explanation for the decrease in bulky PLTs in this study is that enlarged spleens increase the aggregation effect on bulky PLTs. While the alteration in PLT count following training was not statistically significant, it was observed that the decrease in number (6.16) approximated the reduction in the number of bulky PLTs (5.33). An association between reduced PLTs and reduced bulky PLTs was also determined by correlation analysis and a significant correlation was found (*r* = 0.721; *p* = 0.008). In addition, Aster suggested a theory that the possibility of not excluding splenomegaly (causing hypersplenism) inhibits the bone marrow *via* humoral control. Splenomegaly results in an increased aggregation of PLTs while the regenerative capacity of PLTs is inhibited (Aster 1966).

This study found that lymphocytes proliferated in the BH group after 8 weeks of dynamic training and the other immunological parameters remained unaltered. Hypoxia is an environmental stressor that causes the induction of neuroendocrine responses and changes in specific components in the immune system (Facco et al., 2005), including the redistribution of T lymphocytes, a significant reduction in CD4^+^ T-cells and impaired T-cell activation and proliferation (Meehan et al., 1988; Klokker et al., 1993), thereby affecting the human immune function. A combination of exercise and hypoxia results in the effects on immune function being more pronounced than those of exercise or hypoxia independently (Mazzeo, 2005). For example, the combined effect of exercise and hypoxia has a more significant effect on natural killer cells than those that are under normoxia (Klokker et al., 1993). Evidence from cross-sectional studies has shown that a group of Olympic-level athletes noted a decrease in whole blood leukocyte count following 21 days of altitude training (*p* < 0.05) (Pyne et al., 2000). Kitaev and Tokhtabaev, (1981) reported B cells and active immunoglobulin counts to increase following 25 days at high altitude (3,200 m). In another study, no effect on B-cell counts was noted at different altitudes (Facco et al., 2005). Conversely, no change in WBC was observed in this study (*p* = 0.532). However, an increase in lymphocytes from 1.89 ± 0.11 to 2.20 ± 0.13 was observed in the BH group. This parameter remained at the normal range and had no significant effect on the immune function of the athlete, unless the change crossed a critical threshold.

## 6 Conclusion

This is the first study that has investigated the efficacy of 8 weeks of dry dynamic apnoea training on splenic volumes and haematological indices. The study results found dry dynamic breath-hold training to increase spleen volume mong athletes without any alteration to their RBC or HGB levels.

## 7 Limitations

The current study design has several limitations, which should be acknowledged. First, The sample size of our study was small, thus may make the Statistical results underrepresentation. The second criticism concerns the validity of the ultrasonographic assessment of spleen volume. We observed larger spleen volume variation in the breath hold group individuals and reduced spleen volume in control group 2 volunteers (9 ml and 16 ml). Although individual splenic volume and contractility are highly variable in humans (Elia et al., 2021b). It is significant to note that measurement errors associated with ultrasound measurements could have affected these variations (Holmström et al., 2021). Finally, limited by the conditions, only 2-time points were measured, which failed to further eliminate experimental errors.

## Data Availability

The original contributions presented in the study are included in the article/Supplementary Material, further inquiries can be directed to the corresponding authors.
